# IgG4 serologic elevation in a patient with severe hidradenitis suppurativa: a case report and review of the literature

**DOI:** 10.3389/fmed.2024.1471226

**Published:** 2024-10-11

**Authors:** Andrew J. Gauger, Mike Fritz, Callie B. Burgin

**Affiliations:** Department of Dermatology, Indiana University School of Medicine, Indianapolis, IN, United States

**Keywords:** hidradenitis suppurativa, IgG4-related disease, chronic inflammation, laboratory monitoring, immunosuppression, case report

## Abstract

Hidradenitis suppurativa (HS) is a chronic cutaneous and systemic inflammatory condition. Increasingly, reports have found that immunoglobulins play a role in the exaggerated immune response occurring in severe HS. It is important to recognize these implications as HS patients may present with laboratory abnormalities relating to chronic inflammation and immune activation. If these laboratory abnormalities are mistakenly associated with another disease process, it could lead to invasive workup and treatment, causing harm to patients. We describe the case of a 23-year-old woman with Hurley stage III HS who was hospitalized and found to have persistent immunoglobulin-G4 (IgG4) elevation. Upon discharge, the patient was diagnosed with IgG4-related disease (IgG4-RD) and started treatment with azathioprine. However, the biopsy ultimately was negative for IgG4-RD, and she presented to our clinic several months later with worsening HS disease during an active flare. Physical examination revealed actively draining nodules and sinus tracts in the bilateral axillae, inguinal folds, and mons pubis region. A confusing laboratory marker with HS was observed in this case. IgG4 has the potential to inhibit or activate inflammation depending on the context, and so IgG4 elevation has been noted in varying disease states. IgG4 elevation is observed in chronic inflammatory states and may represent a compensatory response by the body. While no other cases have reported the association between HS and IgG4 elevation, IgG levels have been found to reflect HS disease severity. Therefore, IgG4 could play a potential role in HS disease monitoring, and awareness of this association is important for providers when managing patients with HS.

## Introduction

Hidradenitis suppurativa (HS) is a chronic inflammatory process of the skin resulting in high morbidity (1) and profound patient distress (2). It has a prevalence of 1–4% and more commonly occurs in women aged 20–40 years old (2). Approximately 13% of these patients eventually develop severe, Hurley stage III disease, often experiencing rapid disease progression (3). The exact etiology and pathogenesis remain unclear. However, the disease is characterized by follicular occlusion and rupture, followed by a consequent inflammatory response that drives the immune activation necessary for clinical disease progression (4). HS has been associated with various laboratory anomalies reflecting an underlying chronic inflammatory state, including anemia, thrombocytopenia, and leukocytosis. These abnormalities are common in those with severe HS but can normalize with the initiation of immunomodulatory treatment (5). Recently, the immune response in HS has been of great interest to researchers for purposes of understanding disease progression and identifying immune targets for therapy. Increasingly, reports have described elevated immunoglobulins such as IgG in the HS disease process, hinting toward an exaggerated immune response taking place (6–9). Separately, IgG4 has been a recent topic of intense immunologic investigation for its role as a mediator of certain rheumatologic diseases, including IgG4-RD, rheumatoid arthritis (RA), Sjogren’s syndrome, and systemic lupus erythematosus, for instance (10). Herein, we describe the first report of IgG4 elevation in a patient with HS. A review of the literature, including the causes of IgG4 elevation and the role of serum immunoglobulins in HS, is discussed.

## Case presentation

A 23-year-old woman with polycystic ovary syndrome and a 10-year history of Hurley stage III HS was treated for several years with a combination of clindamycin, rifampin, and doxycycline. She was then started on adalimumab for uncontrolled HS. One year later, she presented to her primary care provider with a 1-month history of progressive weight loss, nausea, malaise, and persistent fevers. She was treated for a presumed urinary tract infection with cefdinir.

**Figure 1:**
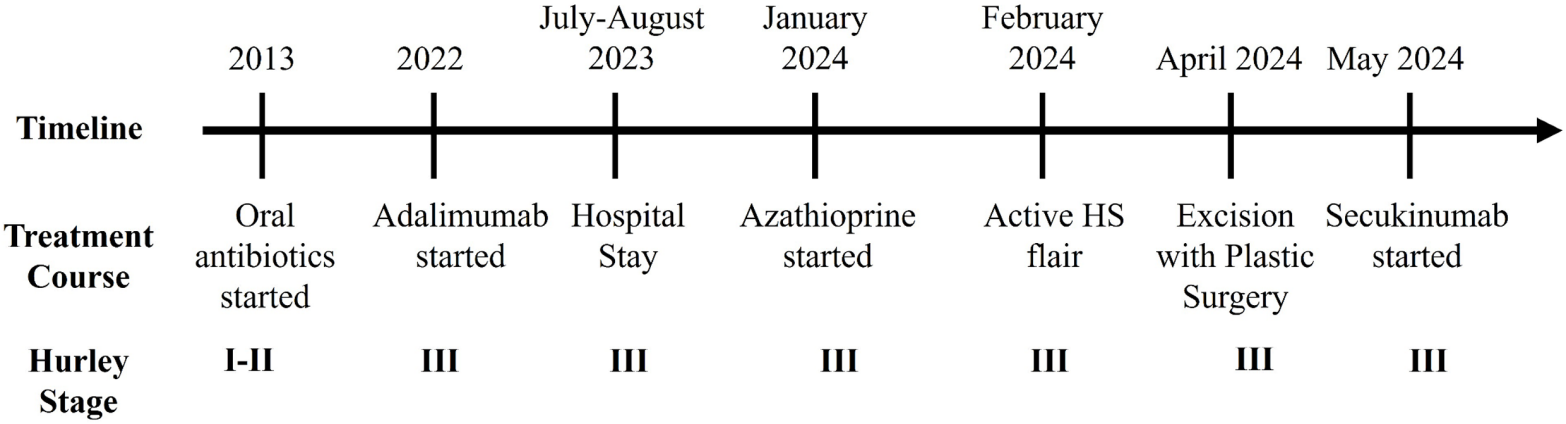
Patient timeline summarizing notable treatment changes and clinical events.

After a lack of improvement, she was then evaluated with a computed tomography (CT), which found heterogeneous enhancement of the kidneys bilaterally, without stranding or abscess. She was subsequently admitted for antibiotic management for treatment of presumed pyelonephritis. However, despite treatment with ceftriaxone and later cefepime, her symptoms did not improve. A repeat CT was unchanged, arguing against pyelonephritis as the source of her fever, fatigue, and other symptoms. In addition to persistent fevers and tachycardia despite antibiotic treatment, she noted jaw swelling on admission, which progressively worsened throughout her hospital stay (Figure 1).

Infectious workup was notable for positive monospot, negative Epstein–Barr virus IgM and polymerase chain reaction (PCR), and positive viral capsid IgGS but was otherwise negative. Rheumatologic workup revealed elevated total IgG and IgG4 serologies (Table 1), along with elevated lysozyme and hypercalcemia. Neck CT imaging was then performed and revealed sialadenitis of the parotids and submandibular glands. Positron emission tomography/computed tomography revealed no evidence of malignant or clonal disease. Instead, it identified hypermetabolic cutaneous thickening of the axillae, inguinal folds, and upper thighs, which was consistent with an infectious or inflammatory process.

**Table 1:** Summary of serum IgA, IgM, IgG, and respective subclass levels in our patient compared to healthy controls (20, 21).

Immunoglobulin class	Patient serologic level (mg/dL)	Normal serologic range in healthy controls
IgA	398	82–470
IgM	90	50–398
IgG (total)	2,752*	694–1760
IgG1	1,104	1,024–1,354
IgG2	1,352*	262–490
IgG3	88	46–100
IgG4	283*	20–51

*Above normal range.

Due to the parotitis and concern for IgG4-RD, cervical lymph node excision and parotid gland biopsies were conducted. Biopsy showed focal chronic sialadenitis and focal fibrosis, and IgG4 immunohistochemical stain of the biopsies revealed that less than 5% of IgG-positive plasma cells were IgG4-positive. Her persistent fevers and jaw swelling resolved by the day of discharge, with no clear underlying cause identified in addition to a positive monospot test. Her adalimumab was held upon admission due to infection concerns, and she was discharged on triple antibiotic therapy with metronidazole, moxifloxacin, and rifampin.

At 1-month follow-up with dermatology, the patient was started on spironolactone, had her antibiotics switched to doxycycline, and received intralesional triamcinolone injections. At a later follow-up with rheumatology, the patient was found to have persistently high IgG4 levels along with elevated lysozyme. Despite a lack of symptoms and non-diagnostic biopsy results for either pathology, the patient was started on azathioprine for suspicion of IgG4-RD vs. sarcoidosis. Six months later, she presented to dermatology in an active HS flare with many draining inflamed nodules and interconnected sinus tracts in axillae, inguinal folds, and mons pubis. Her laboratory results were notable for absolute neutrophilia and a white blood cell count of 18 × 10^9^/L, slightly elevated compared to her hospital admission at 16.7 × 10^9^/L. She received seven injections of intralesional triamcinolone to the nodules, and her antibiotics were switched to levofloxacin for bacterial coverage. She was referred to plastic surgery for urgent debridement of the right axillary skin with a split-thickness skin graft, which was resolved with vacuum-assisted closure and physical therapy. After surgery, secukinumab was started, and spironolactone, rifampin, and clindamycin were continued. At a 3-month follow-up, she presented with a well-healed right axilla and an active flare in the left axilla.

## Discussion

The role of IgG4 across varying disease states is nuanced and a topic of continued investigation. IgG4 has the potential to activate or inhibit inflammation depending on the context of the specific disease state (10). Elevated serum IgG4 has thus been described in a wide range of conditions (11) including autoimmune diseases, cancer, and infections. Discussion of the source of IgG4 elevation is of great importance because management differs depending on the underlying pathology. One report has demonstrated that cancer cells can utilize the inhibitory effect of IgG4 to achieve host immune evasion (12). IgG4 elevation has also been reported in chronic inflammatory states such as RA. Patients with RA and elevated IgG4 out of proportion to total IgG were found to have higher levels of total antibodies, decreased responsiveness to standard RA treatments, and higher disease activity (13). Another report described the association of elevated IgG4 levels with IgG4-RD. This complex clinical condition is characterized by elevated IgG4 levels and infiltration of IgG4-positive plasma cells into organs which drives an inflammatory response. This can present as the enlargement of one or more organs, including the salivary glands, pancreas, and kidney most commonly. In this case, a patient with IgG4 elevation in serum and salivary gland swelling was concerned for the complex IgG4-RD leading to invasive testing for malignancy and heavy immunosuppression. Treatment includes glucocorticoids and adjuvant immunosuppressants, depending on the context (14). Diagnosis primarily relies on tissue biopsy demonstrating typical histopathologic findings combined with an increased tissue presence of IgG4-positive plasma cells. The suggestive morphologic findings on tissue biopsy include storiform fibrosis, dense lymphoplasmacytic infiltrate, and obliterative phlebitis. Elevated IgG4 serologies or IgG4 cell count are both non-specific findings (15). Therefore, excluding other causes of IgG4 elevation is a critically important step in the diagnosis and workup of IgG4-RD.

Although the exact role of IgG in the etiopathogenesis of HS is still being investigated, current research supports that total immunoglobulins are elevated in severe disease (6, 7). The presence of IgG antibodies has been established in both the serum and skin of patients with HS (8, 16). Correlating skin and serum levels of IgG can serve as an accurate predictor of HS disease severity (7, 16) with a threshold of approximately 1,300 mg/mL being predictive of Hurley Stage III disease (7). Our patient had a serum IgG of 2,700 mg/mL that was markedly above the proposed threshold (Table 1). These serologic findings correlated with our clinical observations of her severe Hurley stage III disease. Laboratory analysis demonstrated other known findings of longstanding inflammation in the setting of severe HS, including leukocytosis. In part with the total elevation of the IgG subclass, some degree of IgG4 elevation could thus be expected in the setting of severe HS. Notably, tumor necrosis factor-alpha inhibitors have a variable impact on IgG4 levels (17–19). Long-term treatment with adalimumab and infliximab may result in the development of anti-drug IgG and IgG4 antibodies, and reports have shown that anti-drug IgG4 antibodies are present in 27 and 29% of patients receiving infliximab (17) and adalimumab (18), respectively. Within the context of RA, reports have demonstrated that sustained treatment with adalimumab generally decreased total IgG4 levels (19), so serologic elevation may reflect underlying disease severity. While our patient may have developed anti-adalimumab IgG4 antibodies, it is unclear to what extent these would contribute to her total IgG4 serologic elevation more broadly. Considering the extensive infectious, rheumatologic, and oncologic workups that were negative in our patient, severe HS disease is a reasonable explanation for IgG and IgG4 elevation.

Herein, we describe the first case in the literature of HS associated with elevated IgG4 with no other contributing etiology for the elevated IgG4. Long-term follow-up is needed to monitor how IgG4 levels change over time in our patient on biologic therapy to correlate any decrease or normalization of total IgG and IgG4 with clinical improvement in HS.

## Data Availability

The original contributions presented in the study are included in the article/supplementary material, further inquiries can be directed to the corresponding author.

## References

[1] von der Werth JM Jemec GB. Morbidity in patients with hidradenitis suppurativa. Br J Dermatol. (2001) 144:809–13. doi: 10.1046/j.1365-2133.2001.04137.x 11298541

[2] Seyed Jafari SM Hunger RE Schlapbach C. Hidradenitis suppurativa: current understanding of pathogenic mechanisms and suggestion for treatment algorithm. Front Med. (2020) 7:68. doi: 10.3389/fmed.2020.00068, PMID: 32195261 PMC7064439

[3] Vanlaerhoven AMJD Ardon CB van Straalen KR Vossen ARJV Prens EP van der Zee HH. Hurley III hidradenitis suppurativa has an aggressive disease course. Dermatology. (2018) 234:232–3. doi: 10.1159/000491547, PMID: 30149383 PMC6390447

[4] Napolitano M Megna M Timoshchuk EA Patruno C Balato N Fabbrocini G. Hidradenitis suppurativa: from pathogenesis to diagnosis and treatment. Clin Cosmet Investig Dermatol. (2017) 10:105–15. doi: 10.2147/CCID.S111019 PMC5402905 28458570

[5] Morss-Walton PC Greif C Holcomb ZE Salian P Yankama T Kim D. Treatment of hidradenitis suppurativa resolves associated hematologic abnormalities. Int J Womens Dermatol. (2022) 8:e011. doi: 10.1097/JW9.0000000000000011, PMID: 35620032 PMC9112385

[6] Byrd AS Carmona-Rivera C O'Neil LJ Carlucci PM Cisar C Rosenberg AZ. Neutrophil extracellular traps, B cells, and type I interferons contribute to immune dysregulation in hidradenitis suppurativa. Sci Transl Med. (2019) 11:1–12. doi: 10.1126/scitranslmed.aav5908, PMID: 31484788 PMC11369904

[7] Mintoff D Borg I Pace NP. Serum immunoglobulin G is a marker of hidradenitis suppurativa disease severity. Int J Mol Sci. (2022) 23:13800. doi: 10.3390/ijms232213800, PMID: 36430277 PMC9698525

[8] Hoffman LK Tomalin LE Schultz G Howell MD Anandasabapathy N Alavi A. Integrating the skin and blood transcriptomes and serum proteome in hidradenitis suppurativa reveals complement dysregulation and a plasma cell signature. PLoS One. (2018) 13:e0203672. doi: 10.1371/journal.pone.0203672 30265680 PMC6162087

[9] Frew JW Grand D Navrazhina K Krueger JG. Beyond antibodies: B cells in hidradenitis suppurativa: bystanders, contributors or therapeutic targets? Exp Dermatol. (2020) 29:509–15. doi: 10.1111/exd.14092 32145106

[10] Maslinska M Dmowska-Chalaba J Jakubaszek M. The role of IgG4 in autoimmunity and rheumatic diseases. Front Immunol. (2022) 12:787422. doi: 10.3389/fimmu.2021.787422, PMID: 35145508 PMC8821096

[11] Ebbo M Grados A Bernit E Vély F Boucraut J Harlé JR. Pathologies associated with serum IgG4 elevation. Int J Rheumatol. (2012) 2012:602809. doi: 10.1155/2012/602809 22966232 PMC3433130

[12] Wang H Xu Q Zhao C Zhu Z Zhu X Zhou J. An immune evasion mechanism with IgG4 playing an essential role in cancer and implication for immunotherapy. J Immunother Cancer. (2020) 8:e000661. doi: 10.1136/jitc-2020-000661, PMID: 32819973 PMC7443307

[13] Chen LF Mo YQ Ma JD Luo L Zheng DH Dai L. Elevated serum IgG4 defines specific clinical phenotype of rheumatoid arthritis. Mediat Inflamm. (2014) 2014:635293. doi: 10.1155/2014/635293, PMID: 25548435 PMC4273547

[14] Al-Khalili OM Erickson AR. IgG-4 related disease: an introduction. Mo Med. (2018) 115:253–6. PMID: 30228732 PMC6140155

[15] Deshpande V Zen Y Chan JK Yi EE Sato Y Yoshino T. Consensus statement on the pathology of IgG4-related disease. Mod Pathol. (2012) 25:1181–92. doi: 10.1038/modpathol.2012.72, PMID: 22596100

[16] Carmona-Rivera C O'Neil LJ Patino-Martinez E Shipman WD Zhu C Li QZ. Autoantibodies present in hidradenitis suppurativa correlate with disease severity and promote the release of Proinflammatory cytokines in macrophages. J Invest Dermatol. (2022) 142:924–35. doi: 10.1016/j.jid.2021.07.187, PMID: 34606886 PMC8860851

[17] Vultaggio A Nencini F Carraresi A Pratesi S Movérare R Eriksson C. IgG4 anti-infliximab in treated patients: clinical impact and temporal evolution. Allergy. (2018) 73:2172–81. doi: 10.1111/all.13471, PMID: 29719053

[18] van Schouwenburg PA Krieckaert CL Nurmohamed M Hart M Rispens T Aarden L. IgG4 production against adalimumab during long term treatment of RA patients. J Clin Immunol. (2012) 32:1000–6. doi: 10.1007/s10875-012-9705-0, PMID: 22622790

[19] Sakthiswary R Shaharir SS Wahab AA. Frequency and clinical significance of elevated IgG4 in rheumatoid arthritis: a systematic review. Biomedicine. (2022) 10:558. doi: 10.3390/biomedicines10030558 PMC8945114 35327360

[20] Pellicano C Colalillo A Cusano G Palladino A Pellegrini M Callà CAM. Serum immunoglobulin G (IgG) subclasses in a cohort of systemic sclerosis patients. J Pers Med. (2023) 13:309. doi: 10.3390/jpm13020309, PMID: 36836543 PMC9961548

[21] Gonzalez-Quintela A Alende R Gude F Campos J Rey J Meijide LM. Serum levels of immunoglobulins (IgG, IgA, IgM) in a general adult population and their relationship with alcohol consumption, smoking and common metabolic abnormalities. Clin Exp Immunol. (2008) 151:42–50. doi: 10.1111/j.1365-2249.2007.03545.x, PMID: 18005364 PMC2276914

